# Diversity of Ethiopian Black Cumin (*Nigella sativa* L.) Based on Compositions of Essential Oil

**DOI:** 10.1155/bri/2065593

**Published:** 2025-04-28

**Authors:** Basazinew Degu, Bizuayehu Tesfaye, Wendawek Abebe, Kebebew Assefa

**Affiliations:** ^1^Wondo Genet Agricultural Research Center, Ethiopian Institute of Agricultural Research, P.O. Box 198, Shashemene, Ethiopia; ^2^School of Plant and Horticultural Science, Hawassa University, P.O. Box 05, Hawassa, Ethiopia; ^3^Department of Molecular, Cellular, and Microbial Biology, Addis Ababa University, P.O. Box 345, Addis Ababa, Ethiopia; ^4^Debre Zeit Agricultural Research Centre, Ethiopian Institute of Agricultural Research, P.O. Box 32, Debre Zeit, Ethiopia

**Keywords:** chemotype, essential oil constituents, GC-MS, genotype, *Nigella sativa* L.

## Abstract

Black cumin (*Nigella sativa* L.) seeds essential oil compositions (EOCs) have been used for their medicinal and aromatic values across the world since ancient times. Studies have revealed the presence of variability among black cumin genotypes in EOCs. In Ethiopia, few studies have been conducted to explore the variability of black cumin genotypes by using EOCs. This study investigated the variability of Ethiopian black cumin genotypes (EBCGs) by EOCs. Seeds of 64 *N. sativa* genotypes were used for this experiment. Composite samples of 100 g of seeds were collected and roughly ground from each genotype. The extraction was made by hydrodistillation using a Clevenger-type apparatus for 3 h, and the essential oil was collected by measuring the amount using a measuring pipette. The essential oil samples were stored in a refrigerator at 4°C until gas chromatography–mass spectrometry (GC-MS) analysis. Descriptive statistics was used to estimate the variations among populations' combined mean values of EOCs using the SAS version 9.4 software package. The correlation and hierarchical clustering analysis were made based on the combined mean values of EOCs using the R-software version 4.2.2 packages. A total of 21 EOCs were detected from the essential oil of 64 EBCGs using GC-MS, out of which ρ-cymene, thymoquinone, α-thujene, carvacrol, *trans*-4-methoxythujane, longifolene, terpinen-4-ol, β-pinene, α-pinene, and *d*-limonene dominated the essential oils. Among these, ρ-cymene, thymoquinone, α-thujene, *trans*-4-methoxythujane, and carvacrol were the most abundant constituents in all genotypes, while the rest varied among the genotypes. It is predicted that the major EOCs will be improved by 25.33%–152.14% over improved varieties by selecting the top 5% of landraces. The abundant EOC thymoquinone had a significant and positive correlation with carvacrol and a strong and significant negative correlation with α-thujene, α-pinene, β-pinene, ρ-cymene, and *d*-limonene. Based on the major EOCs, cluster analysis grouped the 64 genotypes into two different chemotypes. Cluster-I: Chemotype A is characterized by a high content of thymoquinone. Cluster-II: Chemotype B is characterized by a high content of ρ-cymene. The presence of the most abundant volatile constituents in genotypes 242835, 9068, and 014_ATH means they are essential for the pharmaceutical and food industries. This study disclosed the existence of a significant diversity of EOCs among the Ethiopian *N. sativa* genotypes, which can be exploited for future improvement programs.

## 1. Introduction

The essential oil of black cumin (*Nigella sativa* L.) contains various bioactive molecules, such as thymoquinone (TQ), thymol, tocopherol, *trans*-retinol, and selenium [1]. It is used worldwide for functional foods and nutraceuticals [2]. According to Javed et al. [2], black cumin is a remarkable plant that has been referred to as “the herb from heaven” by early herbalists. Medicinally, it is used for many complaints. TQ and nigellimine are among the main components [3]. The oil also treats respiratory conditions such as asthma, emphysema, and bronchitis [4]. Several findings reported that black cumin has different pharmacological activities such as antioxidant [5], antidiabetic [6], antifungal [7–11], antiviral [12–15], antiparasitic [16–19], anticancer [20], and antibacterial [21].

The variability of *N. sativa* genotype based on essential oil volatile compounds was reported by different authors from different countries [22–24]. TQ was reported as the first main component of Egyptian, Turkish, and Chinese *N. sativa* seeds essential oils [22–24]. In addition, ρ-cymene was reported as the first main component of the three Ethiopian *N. sativa* varieties of seeds essential oil [25]. TQ has been used worldwide for its biological activities among the main components of *N. sativa* seeds essential oil with the highest share.

For the past 3000 years, *N. sativa* has been cultivated in Ethiopia. Currently, it has been widely distributed in the Oromia region (Bale and Arsi Zone), Amhara region (East Gojjam, West Gojjam, North Gondar, South Gondar, and North Wollo and South Wollo zones), Tigray region (central, western and northwestern zones), Benishangul-Gumuz region (Metekel and Asosa zones), and Southwest Ethiopia Peoples' Region (Keficho Shekicho zone) of Ethiopia. It has been used as a flavor in bread and sauces, as well as an ingredient in the “berbere” spice mix.

In Ethiopia, whole seeds were used for a long period, and fixed oil (oleoresin) of *N. sativa* has been used recently for its spicy and medicinal values. However, the variability of some of the existing genotypes was made by their compositions of essential oil [25]. However, more genotypes were not involved during characterization. This would hinder the potential of the genotypes to be exploited for future breeding and improvement programs. Therefore, this study was designed to investigate the variability of Ethiopian *N. sativa* genotypes by their essential oil compositions. The significance of this study was to determine the variability of the genotypes used and provide information for their conservation, improvement, and utilization.

## 2. Materials and Methods

### 2.1. Genotypes Collection

The matured dried seeds of 64 *N. sativa* genotypes (eight improved varieties and 56 accessions) harvested at a moisture content of 11% from the field experiments conducted at Debre Zeit and Kulumsa Agricultural Research Centers experimental fields stored in seed store by putting them in paper bags were used for this experiment (Table 1).

### 2.2. Sample Preparation and Extraction of Essential Oil by Hydrodistillation

Composite samples of 100 g of *N. sativa* seeds were collected and roughly ground from each genotype. Extraction was made by hydrodistillation using a Clevenger-type apparatus for 3 h, and the essential oil was collected by measuring the amount using a measuring pipette. The essential oil samples were stored in a refrigerator at 4°C until gas chromatography–mass spectrometry (GC-MS) analysis.

### 2.3. GC-MS Analysis of Essential Oil

The collected essential oil was dried with anhydrous Na_2_SO_4_, and 0.2% (w/v) of the essential oil solution was prepared by diluting in n-hexane, and the instrument was conditioned with a split less injector mode. To identify the composition of essential oil, GC-MS analyses were performed on an Agilent GC model 7820A series equipped with an autosampler (Agilent model G4513A), coupled to a mass spectrometer detector (Agilent model 5975 series) quadrupole analyzer operating in the electron ionization (EI) mode at 70 ev. Helium was used as carrier gas (1.5 mL/min) with a capillary column of HP-5MS (30 m × 0.25 mm id; 0.25 μm film thickness). The GC-MS transfer line temperature was set at 200°C. The column temperature program was the same as that used for the GC analyses. The inlet and ionization source temperatures were set at 250°C and 160°C, respectively. Electron-impact mass spectra were recorded over the range of 40–450 amu at 0.5 scan. The injected volume was 1 μL of the oil. The GC-MS run time for each genotype was 40 min, with a solvent delay time of 2 min. The qualitative and quantitative composition of the essential oils was ascertained using GC-MS. Qualitatively, Wei et al. [26] identified the components by comparing them with the NIST-11 database and using the retention index. Quantitatively, the peak area–approximated peak height approximation was used to express the relative amounts of the constituents of the essential oils as a percentage [27].

### 2.4. Data Analysis

Descriptive statistics (mean and range) were used to estimate the variations among populations combined mean values of essential oil compositions using the SAS Version 9.4 software package [28]. The combined mean values of major volatile compounds were subjected to bivariate analysis (i.e., correlation analysis) and multivariate analysis (i.e., cluster analysis) methods using R-software Version 4.2.2 [29].

Hierarchical cluster analysis was performed by using R-software packages, “class” [30], “clv” [31], “factoextra” [32], and “cluster” [33]. Clustering was used to examine the aggregation patterns of the 64 black cumin genotype populations and the six groups of genotypes collected based on their similarity concerning the corresponding means of all the volatile compounds that were collected. The optimum number of clusters for the data set was determined by using the Silhouette method in the R-software package [29]. The genetic distance was measured using Euclidean distance, and the distance matrix was used to construct the dendrograms using Ward's minimum variance method [34].

## 3. Results and Discussion

### 3.1. Chemical Composition of *N. sativa* Essential Oils

**Figure 1:**
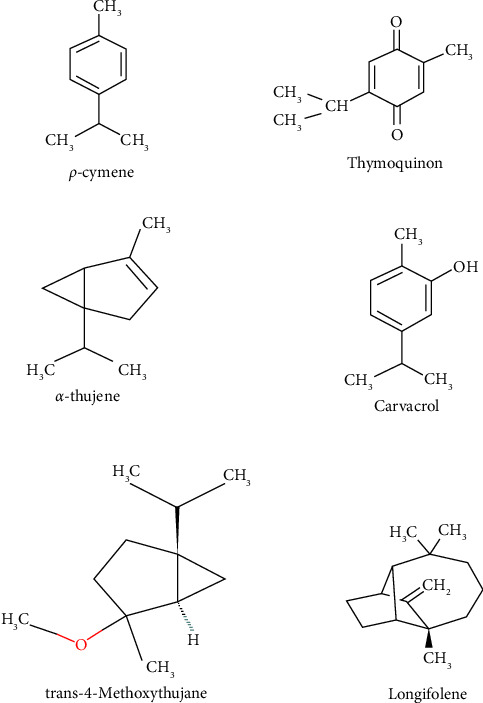
Chemical structures of the six most abundant volatile compounds of *N. sativa* L. essential oils from Ethiopia.

Essential oils extracted from 64 black cumin genotypes were characterized by GC-MS, allowing for the identification of 21 volatile compounds, of which 10 are major essential oil compounds (Tables 2 and 3). So far 19, 31, 85, 22, 113, 48, 20, and 40 volatile compounds were detected in Egyptian [22], Chinese [24], Ethiopian [25], Italian [35], Algerian [36], Polish [37], Tunisian [38], and Iranian [39] *N. sativa*, respectively. The major volatile compounds identified in most of the genotypes were ρ-cymene (23.93%–45.61%), TQ (14.81%–42.44%), α-thujene (0.91%–12.97%), carvacrol (3.01%–11.48%), *trans*-4-methoxythujane (4.82%–11.29%), longifolene (2.1%–6.97%), terpinen-4-ol (0%–3.34%), β-pinene (0%–2.82%), α-pinene (0%–2.75%), and *d*-limonene (0.37%–1.86%) (Table 2). Of the 10 major compounds, six of them (Figure 1) occur in more than 6% of the content. The six compounds: ρ-cymene (45.61%), TQ (42.44%), α-thujene (12.97%), carvacrol (11.48%), *trans*-4-methoxythujane (11.29%), and longifolene (6.97%) were the most abundant volatile constituents found in all genotypes (Figure 1 and Table 3). Out of them, TQ is the most important bioactive compound for phytomedicinal values. The highest TQ content (67.7% and 63.3%) was reported by Palabiyik and Aytac [23] and Edris [22] from Turkish and Egyptian *N. sativa*, respectively. *N. sativa* essential oil is used for functional foods and nutraceuticals/pharmaceuticals [40]. Thus, Ethiopia is the third most important country next to Turkey and Egypt because of its rich potential of *N. sativa* with the highest TQ content to be exploited for functional foods and pharmaceuticals.

Pico-cymene was the first dominant constituent ranging from 23.93% (9068) to 45.61% (242221) across genotypes with an average of 35.16%. Six genotypes, 242221 (45.61%), 8502 (45.3%), 9069 (44.44%), 242825 (42.63%), 242839 (42.52%), and 90510 (40.71%), recorded more than 40% ρ-cymene content (Table 3). The result is in line with the report of Nickavar et al. [41] and Rezaei-Chiyaneh et al. [39], who reported that one of the major volatile constituents of Iranian *N. sativa* seeds was ρ-cymene (14.80% and 15.59%, respectively). Similarly, D'Antuono et al. [35], Benkaci–Ali et al. [36], Wajs et al. [37], Palabıyık and Aytaç [23], Ramadan [42], Abera and Hirko [25], and Farhan et al. [43] reported ρ-cymene as one of the major volatile constituents of *N. sativa* seeds. ρ-Cymene is an important volatile constituent used in pharmaceutical industries for the production of anticancer [44], anti-inflammatory [45], antimicrobial [46], and antioxidant [47, 48] drugs.

The second most abundant constituent was TQ, ranging from 14.81% (8502) to 42.44% (242835) across genotypes with an average of 31.08%. Four genotypes, 242835 (42.44%), 9068 (41.14%), 242841 (40.35%), and 014_ATH (40.01%) recorded more than 40% TQ content (Table 3). Authors from different countries reported TQ among the main constituents in the essential oil of *N. sativa* seeds [22–25, 35, 36, 38, 42, 43, 49]. Traditionally, TQ has been reported as a natural drug [50]. Chao et al. [51] reported TQ as an effective natural antimicrobial preservative that has potential applications in regulating food contamination and foodborne diseases caused by *Bacillus cereus*. TQ is an important volatile constituent used in pharmaceutical industries for the production of antibacterial, antioxidant [38], inflammatory [52], anticancer [53], hepatoprotective [54], antidiabetic [55], anti–fertility-enhancing [56, 57], analgesic [58], and antimicrobial [46] drugs.

Alpha-thujene was the third dominant constituent ranging from 0.91% (9068) to 12.97% (242221) across genotypes with an average of 5.76%. Six genotypes, 242221 (12.97%), 90501 (11.99%), 012_ATH (11.13%), 90510 (10.62%), 229808 (10.42%), and 90504 (10.41%), recorded more than 10% α-thujene content (Table 3). This result is in line with the findings of Bourgou et al. [38], Ramadan [42], and Abera and Hirko [25], who reported α-thujene as one of the major volatile constituents from Turkish, Egyptian, and Ethiopian *N. sativa* seeds, respectively. α-Thujene is an important volatile constituent used in pharmaceutical industries for the production of antimicrobial [59] drugs.

The fourth abundant constituent was carvacrol, ranging from 3.01% (90504) to 11.48% (242838) across genotypes with an average of 5.91%. Four genotypes, 242838 (11.48%), Silingo (10.08%), 007_ATH (9.75%), and 9068 (9.23%) recorded more than 9% carvacrol content (Table 3). Supporting results were reported by Benkaci–Ali et al. [36], Ramadan [42], Palabıyık and Aytaç [23], and Rezaei-Chiyaheh et al. [39] from Algeria, Egypt, Turkey, and Iran with respective values of 12.9%, 2.12%, 8.40%, and 4.65%. Rattanachaikunsopon and Phumkhachorn [60] reported a good inhibitory effect of carvacrol against *Vibrio cholerae*. Carvacrol is an important volatile constituent used in pharmaceutical industries for the production of antimicrobial [46] and antioxidant [61] drugs.

The fifth dominant constituent was *trans*-4-methoxythujane, ranging from 4.82% (242844) to 11.29% (242825) across genotypes with an average of 8.78%. Ten genotypes: 242825 (11.29%), 9068 (11.22%), 007_ATH (10.98%), 003_ATH (10.93%), 242838 (10.70%), 015_ATH (10.53%), 019_ATH (10.34%), 004_ATH (10.31%), 009_ATH (10.16%), and 010_ATH (10.07%), recorded more than 10% *trans*-4-methoxythujane content (Table 3). This result agrees with the finding of Abera and Hirko [25], who reported that one of the major volatile constituents of Ethiopian *N. sativa* seeds was *trans*-4-methoxythujane (8.86%).

The sixth dominant constituent was longifolene, ranging from 2.1% (90501) to 6.97% (004_ATH) across genotypes with an average of 3.67%. Seven genotypes, 004_ATH (6.97%), 242835 (6.27%), 019_ATH (5.99%), 003_ATH (5.86%), 9068 (5.75%), 242838 (5.52%), and 007_ATH (5.42%), recorded more than 5% longifolene content (Table 3). Supporting results were reported by Ramadan [42] and Abera and Hirko [25] from Egypt and Ethiopia, respectively. Another important volatile constituent, longifolene, is used in pharmaceutical industries for the manufacture of antibacterial [38] drugs.

The essential oil of *N. sativa* seeds has different biological activities due to the presence of various kinds of volatile compounds. Among the major volatile compounds, TQ is used for antioxidant, antiparasitic, hepatoprotective, antidiabetic, analgesic, anticancer, antimicrobial, anti-inflammatory, antibacterial, and fertility-enhancing activities. Another major volatile compound is carvacrol, which has antioxidant and antimicrobial activity. Furthermore, *p-cymene* and longifolene have antimicrobial and antibacterial activity, respectively.

The wide range of major volatile compounds witnessed the presence of variability among the black cumin genotypes (Tables 2 and 4). This showed that there is a possibility of improving these traits through selection. Hence, the selection of the top 5% of the genotype is expected to improve biochemical traits by 13.16%–152.14% through selection (Table 4). Based on this, genotypes 242835 (from Oromia), 9068, and 014_ATH (from Amhara) were the top 5% best-performed landraces over improved varieties selected for the production of major volatile compounds (Table 3). Therefore, they can be directly promoted for the production of those major volatile compounds.

### 3.2. Relationship Among Volatile Compounds

**Figure 2:**
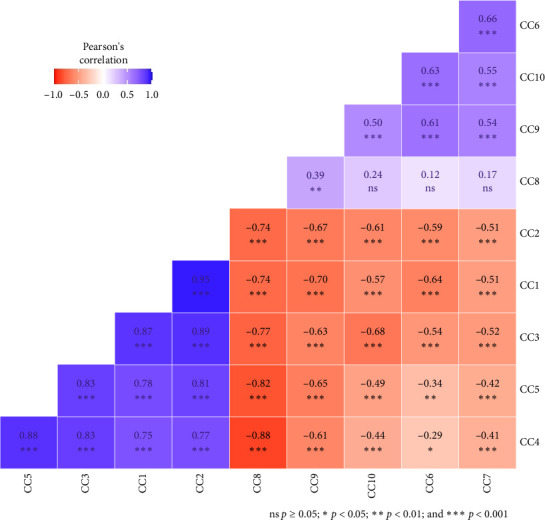
The correlation coefficient of the 10 major volatile compounds of 64 *N. sativa* L. genotypes of Ethiopia (Note: CC = chemical compound: CC1 = α-thujene, CC2 = α-pinene, CC3 = β-pinene, CC4 = ρ-cymene, CC5 = *d*-limonene, CC6 = *trans*-4-methoxythujane, CC7 = terpinen-4-ol, CC8 = thymoquinone, CC9 = carvacrol, and CC10 = longifolene).

A correlation analysis was made among the 10 major volatile compounds to determine the degree and direction of the relationship among them (Figure 2). Alpha-thujene had a strong positive and significant correlation with α-pinene (*r* = 0.95^∗∗∗^), β-pinene (*r* = 0.87^∗∗∗^), *d*-limonene (*r* = 0.78^∗∗^), and ρ-cymene (*r* = 0.75^∗∗∗^) and negatively correlated with TQ (*r* = −0.74^∗∗∗^), carvacrol (*r* = −0.70^∗∗∗^), *trans*-4-methoxythujane (*r* = −0.64^∗∗∗^), longifolene (*r* = −0.57^∗∗∗^), and terpinen-4-ol (*r* = −0.51^∗∗∗^). The significant positive correlation of α-thujene with α-pinene, β-pinene, *d*-limonene, and ρ-cymene indicated that the higher the genotype with α-pinene, β-pinene, *d*-limonene, and ρ-cymene, the higher the α-thujene content. They have a direct relationship. Therefore, those compounds can be improved simultaneously. The major volatile chemical constituent ρ-cymene correlated positively with *d*-limonene (*r* = 0.88^∗∗∗^) and negatively with TQ (*r* = −0.88^∗∗∗^), carvacrol (*r* = −0.61^∗∗∗^), longifolene (*r* = −0.44^∗∗∗^), and terpinen-4-ol (*r* = −0.41^∗∗∗^). TQ had a positive correlation with longifolene (*r* = 0.24), terpinen-4-ol (*r* = 0.17), and *trans*-4-methoxythujane (*r* = 0.12); a significant positive correlation with carvacrol (*r* = 0.39^∗∗^); and a significant negative correlation with ρ-cymene (*r* = −0.88^∗∗∗^), *d*-limonene (*r* = −0.88^∗∗∗^), β-pinene (*r* = −0.77^∗∗∗^), α-thujene (*r* = −0.74^∗∗∗^), and α-pinene (*r* = −0.74^∗∗∗^). The significant negative correlation of TQ with ρ-cymene, *d*-limonene, β-pinene, α-thujene, and α-pinene indicated that the higher the genotype with ρ-cymene, *d*-limonene, β-pinene, α-thujene, and α-pinene content, the lower the content of TQ. This implied that those traits cannot be improved simultaneously. However, it is possible in the case of TQ and carvacrol.

### 3.3. Cluster Analysis

**Figure 3:**
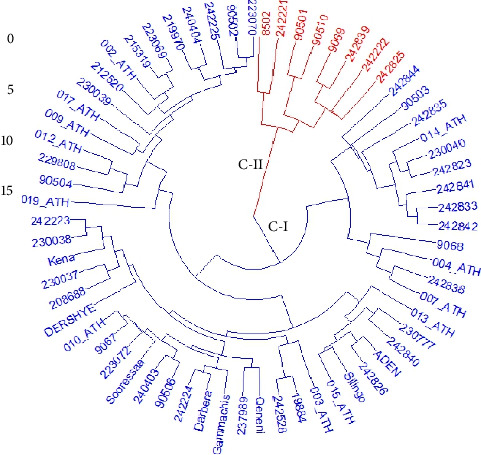
Phylogram showing the relationship between the 64 *N. sativa* genotypes of Ethiopia based on the 10 major volatile compounds using the Euclidean similarity index (blue and red colors represent Clusters I and II, respectively).

Cluster analysis was done based on 10 major volatile compounds of the 64 *N. sativa* genotypes of Ethiopia to examine the extent and pattern of the genotypes based on their similarity. The optimum number of clusters determined for the data set was two. Based on this, the average clustering method was used to classify the 64 *N. sativa* genotypes of Ethiopia into two different clusters (Figure 3). The number and name of genotypes in each cluster with their collection regions were presented in Table 1.

Cluster I was the largest group, having 56 (87.5%) genotypes comprised from all collection regions and all of the improved varieties (Figure 3 and Table 5). The entire genotypes from the Tigray, SNNP, and Benishangul-Gumuz regions were clustered under this group. The group was characterized by a significant content of TQ, which represents Chemotype A (Table 6). Genotype 242835 (from Oromia) contained a considerably high content of TQ (42.44%), followed by 9068 (41.14%) and 014_ATH (40.35%) (Table 3). Genotypes better fitted for the production of pharmaceutical products may be identified through selection in this group by considering its chemotype.

**Figure 4:**
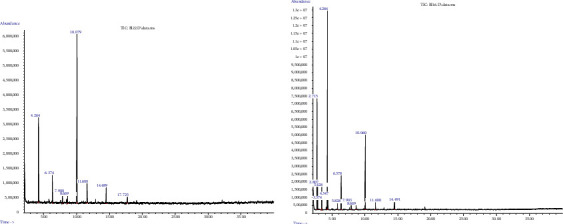
GC-MS chromatogram of *N. sativa* essential oils of Ethiopia for each cluster.

Cluster II contained eight (12.5%) genotypes from the Amhara and Oromia regions (Figure 3 and Table 5) and is characterized by the higher mean value of ρ-cymene, which represents Chemotype B (Table 6). Genotype 242221 (from Amhara) contained a considerably high content of ρ-cymene (45.61%), followed by 8502 (45.30%) and 9069 (10.98%) (Table 3). Chromatograms of the compounds representing the different clusters are shown in Figure 4.

### 3.4. Volatile Compounds' Diversity in Groups of Genotypes

The genotypes from the Amhara and Oromia regions were spread into the two clusters but in different proportions (Table 7). However, all genotypes from the Benishangul-Gumuz, Tigray, and SNNP regions and improved varieties were not spread into the two clusters but rather grouped under the first cluster.

## 4. Conclusions

Sixty-four *N. sativa* genotypes of essential oil were analyzed using GC-MS, and a total of 27 compounds were identified. The results indicated that the essential oils were dominated by ρ-cymene, TQ, α-thujene, carvacrol, *trans*-4-methoxythujane, longifolene, terpinen-4-ol, β-pinene, α-pinene, and *d*-limonene. Five compounds (ρ-cymene, TQ, α-thujene, *trans*-4-methoxythujane, and carvacrol) were the most abundant constituents in all genotypes, while the rest varied among the genotypes. It is expected to improve all major volatile compounds by 25.33%–152.14% over improved varieties through the selection of the top 5% landraces. TQ had a significant and positive correlation with carvacrol and a strong and significant negative correlation with α-thujene, α-pinene, β-pinene, ρ-cymene, and *d*-limonene. Cluster analysis grouped the 64 genotypes into two chemotypes. Chemotype A has a high TQ content, while Chemotype B has a high ρ-cymene concentration. The discovered high volatile constituent variabilities demonstrated considerable diversity across the 64 Ethiopian *N. sativa* genotypes, which can be used in future breeding and improvement programs. The most abundant volatile constituents, TQ and ρ-cymene, are highly applicable to pharmaceutical and nutraceutical industries. Therefore, genotypes 242835, 9068, and 014_ATH are promising for pharmaceutical and food industries due to the presence of the most important volatile constituents. This finding provided baseline information for the beneficiaries to conserve, utilize, and improve the genotypes.

## Figures and Tables

**Table 1:** Lists of black cumin (*N. sativa* L.) genotypes with their area of collection in Ethiopia.

Genotype	Region	Zone	Latitude (°N)	Longitude (°E)	Source	Status
Darbera	Oromia	Bale	—	—	SARC	Improved
DERSHYE	Oromia	Bale	—	—	SARC	Improved
ADEN	Oromia	East Shewa	—	—	DZARC	Improved
Sooressaa	Oromia	Bale	—	—	SARC	Improved
Gammachis	Oromia	Bale	—	—	SARC	Improved
Silingo	Oromia	East Shewa	—	—	DZARC	Improved
Kena	Oromia	Bale	—	—	SARC	Improved
Qeneni	Oromia	Bale	—	—	SARC	Improved
8502	Oromia	Bale	7.0000	39.8000	DZARC	Accession
9067	Amhara	West Gojjam	11.6856	37.0200	DZARC	Accession
9068	Amhara	West Gojjam	11.7611	37.0844	DZARC	Accession
9069	Amhara	West Gojjam	10.6467	37.0858	DZARC	Accession
19884	SNNP	Keficho Shekicho	7.0644	36.0667	DZARC	Accession
90501	Amhara	West Gojjam	10.6392	37.0869	DZARC	Accession
90502	Amhara	South Gondar	11.9500	37.7000	DZARC	Accession
90503	Amhara	South Gondar	11.9833	37.7667	DZARC	Accession
90504	Oromia	Arsi	8.0500	38.7833	DZARC	Accession
90506	Amhara	East Gojjam	10.3333	38.0000	DZARC	Accession
90510	Oromia	West Shewa	9.1667	37.8333	SARC	Accession
208688	Oromia	West Hararghe	8.8167	40.4167	SARC	Accession
212520	Oromia	Bale	7.0167	39.9833	SARC	Accession
215319	Amhara	East Gojjam	11.0022	37.0031	SARC	Accession
219970	Tigray	Western	14.1367	38.3094	DZARC	Accession
223069	Amhara	East Gojjam	11.0022	37.0031	SARC	Accession
223070	B/Gumuz	Metekel	11.0000	35.7625	SARC	Accession
223072	B/Gumuz	Metekel	11.0000	35.7625	DZARC	Accession
229808	B/Gumuz	Metekel	10.5000	36.1667	DZARC	Accession
230037	Tigray	Central	14.0667	38.0833	SARC	Accession
230038	Tigray	Central	14.1667	38.7500	SARC	Accession
230039	Tigray	Northwestern	14.0667	38.0833	DZARC	Accession
230040	Tigray	Central	14.0833	39.1000	SARC	Accession
230777	Oromia	Borena	5.1167	39.4833	SARC	Accession
237989	Oromia	Bale	8.0500	38.7833	SARC	Accession
240403	SNNP	Keficho Shekicho	7.2342	35.7092	SARC	Accession
240404	SNNP	Keficho Shekicho	7.2500	36.0000	SARC	Accession
242221	Amhara	South Wollo	10.8411	39.8167	DZARC	Accession
242222	Amhara	North Wollo	11.9681	39.0722	DZARC	Accession
242223	Tigray	Western	14.1208	38.4747	DZARC	Accession
242224	SNNP	Arbaminch	6.1186	38.1186	SARC	Accession
242225	Amhara	South Wollo	11.0333	39.7542	DZARC	Accession
242528	B/Gumuz	Asosa	9.9858	34.6675	DZARC	Accession
242825	Oromia	Arsi	7.5606	39.6081	DZARC	Accession
242826	Oromia	Arsi	7.5744	39.5969	DZARC	Accession
242833	Oromia	Arsi	7.6508	39.4961	DZARC	Accession
242835	Oromia	Arsi	7.6036	39.5636	DZARC	Accession
242838	Oromia	Arsi	7.6031	39.5414	DZARC	Accession
242839	Oromia	Arsi	7.5719	39.5283	DZARC	Accession
242840	Oromia	Arsi	7.5544	39.5333	DZARC	Accession
242841	Oromia	Arsi	7.5511	39.5408	DZARC	Accession
242842	Oromia	Arsi	7.5356	39.5364	DZARC	Accession
242844	Oromia	Arsi	7.6061	39.5233	DZARC	Accession
242823	Oromia	Arsi	7.6036	39.5619	DZARC	Accession
002_ATH	Amhara	North Gondar	12.3129	37.3220	DZARC	Accession
003_ATH	Amhara	North Gondar	12.3401	37.3688	DZARC	Accession
004_ATH	Amhara	North Gondar	12.3522	37.3389	DZARC	Accession
007_ATH	Amhara	North Gondar	12.4253	37.2989	DZARC	Accession
009_ATH	Amhara	North Gondar	12.2181	37.1950	DZARC	Accession
010_ATH	Amhara	North Gondar	12.3575	37.1872	DZARC	Accession
012_ATH	Amhara	North Gondar	12.3417	37.1092	DZARC	Accession
013_ATH	Amhara	North Gondar	12.3072	37.3158	DZARC	Accession
014_ATH	Amhara	North Gondar	12.2475	37.0406	DZARC	Accession
015_ATH	Amhara	North Gondar	12.2300	37.0325	DZARC	Accession
017_ATH	Amhara	North Gondar	12.1522	37.0150	DZARC	Accession
019_ATH	Amhara	South Wollo	11.2636	39.6803	DZARC	Accession

Abbreviations: B/Gumuz = Benishangul-Gumuz, DZARC = Debre Zeit Agricultural Research Center, and SARC = Sinana Agricultural Research Center.

**Table 2:** Retention index, mean, and range of chemical compounds (%) identified across 64 *N. sativa* L. genotypes of Ethiopia at each testing site and pooled during the 2021 cropping season.

No.	Chemical compounds	RI	Debre Zeit	Kulumsa	Combined	
			Mean ± SE	Mean ± SE	Mean ± SE	Range
1	α-Thujene	924	5.69 ± 0.45	5.82 ± 0.53	5.76 ± 0.33	0.91–12.97
2	α-Pinene	932	1.30 ± 0.11	1.18 ± 0.12	1.24 ± 0.07	0–2.75
3	Bicyclo[3.1.0]hexane, 4-methylene-1-(1-methylethyl)-	964	0.73 ± 0.06	0.50 ± 0.07	0.61 ± 0.05	0–1.35
4	β-Pinene	974	1.68 ± 0.11	1.56 ± 0.09	1.62 ± 0.07	0–2.82
5	(+)-4-Carene	1022	—	0.14 ± 0.04	0.07 ± 0.02	0–0.6
6	Eucalyptol	1023	—	0.01 ± 0.01	0.01 ± 0.01	0–0.47
7	*d*-Limonene	1024	1.22 ± 0.06	1.01 ± 0.07	1.11 ± 0.05	0.37–1.86
8	ρ-Cymene	1042	31.76 ± 0.79	38.56 ± 0.75	35.16 ± 0.56	23.93–45.61
9	γ-Terpinene	1054	0.02 ± 0.02	0.72 ± 0.19	0.37 ± 0.09	0–2.93
10	α-Thujone	1101	—	0.08 ± 0.05	0.04 ± 0.02	0–1.13
11	*trans*-4-Methoxythujane	1120	8.51 ± 0.18	9.05 ± 0.24	8.78 ± 0.15	4.82–11.29
12	Terpinen-4-ol	1159	1.68 ± 0.10	1.08 ± 0.12	1.38 ± 0.08	0–3.34
13	2-(Octyloxycarbonyl)benzoic acid	1160	0.04 ± 0.03	0.04 ± 0.03	0.04 ± 0.02	0–0.87
14	Camphor	1192	—	0.06 ± 0.04	0.03 ± 0.02	0–0.75
15	Thymoquinone	1248	37.15 ± 1.24	25.01 ± 1.05	31.08 ± 0.80	14.81–42.44
16	Carvacrol	1278	3.44 ± 0.17	8.37 ± 0.44	5.91 ± 0.24	3.01–11.48
17	Thymol	1290	—	0.17 ± 0.09	0.08 ± 0.04	0–2.09
18	α-Longipinene	1347	0.07 ± 0.04	0.02 ± 0.02	0.05 ± 0.02	0–1.22
19	Longifolene	1408	3.68 ± 0.18	3.65 ± 0.16	3.67 ± 0.13	2.1–6.97
20	Humulene	1447	—	0.03 ± 0.03	0.01 ± 0.01	0–0.84
21	Naphthalene, decahydro-4a-methyl-1-methylene-7-(1-methylethylidene)-, (4aR-*trans*)-	1470	—	0.16 ± 0.10	0.08 ± 0.05	0–2.72

Abbreviations: RT = retention index and SE = standard error.

**Table 3:** The 10 major volatile chemical compounds (%) of 64 *N. sativa* genotypes of Ethiopia across two locations during 2021 cropping seasons.

Genotype	EOC (%)	Chemical compound (%)									
		CC1	CC2	CC3	CC4	CC5	CC6	CC7	CC8	CC9	CC10
Darbera	0.40	5.10	1.08	1.43	34.74	0.95	9.31	1.62	32.58	6.60	3.28
DERSHYE	0.33	6.91	1.57	1.77	32.44	1.32	8.55	1.58	30.58	6.53	3.71
ADEN	0.43	4.42	1.20	1.50	31.38	1.08	9.27	1.59	33.99	8.59	3.49
Sooressaa	0.31	4.40	1.08	1.57	34.18	1.21	9.41	─	35.07	5.42	3.47
Gammachis	0.38	5.70	1.44	1.86	35.92	0.74	9.40	0.70	31.54	6.90	2.88
Silingo	0.18	3.65	0.67	1.36	30.53	0.62	9.74	1.66	32.75	10.08	3.17
Kena	0.30	7.06	1.14	2.07	37.11	0.68	7.32	0.64	32.03	4.68	2.91
Qeneni	0.35	5.39	1.25	1.72	34.58	0.98	7.77	1.56	36.40	4.38	2.47
8502	0.40	9.45	2.03	2.67	45.30	1.76	9.27	1.32	14.81	5.65	3.40
9067	0.38	4.26	1.16	1.53	34.12	1.04	9.11	1.51	34.50	5.88	3.86
9068	0.23	0.91	─	─	23.93	0.37	11.22	2.62	41.14	9.23	5.75
9069	0.33	8.04	1.64	2.00	44.44	1.86	8.16	0.87	20.71	4.11	3.27
19884	0.25	3.95	0.98	1.41	35.01	1.02	9.20	0.94	30.58	8.70	3.78
90501	0.55	11.99	2.75	2.61	38.53	1.56	6.09	0.66	21.68	3.79	2.10
90502	0.47	7.02	1.53	2.05	39.87	1.47	7.85	0.76	28.12	3.70	2.98
90503	0.30	4.57	0.81	1.45	34.46	1.12	7.52	─	40.94	4.27	2.82
90504	0.55	10.41	2.11	2.23	36.42	1.37	6.98	0.56	29.44	3.01	2.13
90506	0.33	5.34	1.25	1.68	33.04	1.01	8.76	1.53	34.07	5.84	3.86
90510	0.35	10.62	2.21	2.38	40.71	1.53	7.93	0.98	21.37	3.67	2.62
208688	0.24	7.12	1.14	1.84	35.80	1.25	7.29	0.86	30.13	5.08	2.42
212520	0.29	8.19	1.47	2.25	38.89	1.50	8.64	1.37	24.59	7.03	3.58
215319	0.54	7.30	1.50	2.04	39.90	1.48	7.96	0.96	26.24	4.28	3.40
219970	0.49	7.54	1.59	2.00	37.16	1.37	9.51	1.74	27.15	4.25	3.23
223069	0.41	7.12	1.57	2.14	39.52	1.41	8.90	1.48	26.03	4.54	3.46
223070	0.38	8.28	1.86	2.23	39.10	1.39	8.38	0.72	28.64	4.42	2.42
223072	0.45	4.40	1.05	1.56	35.33	1.01	9.16	1.57	34.37	5.09	3.31
229808	0.53	10.42	1.99	2.25	36.21	1.43	7.25	1.01	25.88	3.61	3.25
230037	0.43	8.07	1.75	2.25	36.16	1.38	8.12	0.94	28.51	4.50	3.33
230038	0.40	6.66	1.69	1.91	34.35	1.23	8.03	0.85	32.32	5.22	2.87
230039	0.35	6.90	1.53	1.79	35.76	1.30	8.20	0.90	25.96	6.03	4.49
230040	0.40	3.80	0.73	0.97	29.80	0.69	9.61	1.81	38.00	7.71	3.75
230777	0.25	3.62	0.93	1.23	32.08	0.68	9.32	1.76	36.93	7.69	3.53
237989	0.35	4.60	1.26	1.62	35.49	1.27	7.74	1.02	34.08	4.08	2.62
240403	0.40	5.64	1.57	1.70	33.26	1.23	9.35	1.90	34.30	5.35	2.66
240404	0.18	8.26	1.58	1.68	37.80	1.24	9.20	2.02	28.29	3.50	4.16
242221	0.27	12.97	2.66	2.82	45.61	1.68	6.82	0.74	16.61	3.36	3.09
242222	0.39	7.29	1.78	1.44	39.66	1.43	9.65	1.69	20.82	7.51	3.59
242223	0.45	6.50	1.68	1.89	34.99	1.36	7.37	1.14	33.11	4.64	2.46
242224	0.35	4.74	1.10	1.72	35.61	1.16	8.88	1.47	32.93	5.58	3.01
242225	0.45	6.00	1.37	1.80	38.89	1.36	8.82	1.41	29.98	4.15	3.12
242528	0.38	4.53	1.23	1.54	35.80	0.84	9.11	1.55	29.94	8.45	4.31
242825	0.28	5.58	1.39	2.06	42.63	1.54	11.29	1.73	18.38	6.66	4.56
242826	0.40	5.24	1.26	1.71	32.32	0.99	8.82	1.83	33.29	7.58	3.57
242833	0.30	3.44	0.46	1.11	31.39	0.95	8.90	1.69	39.62	5.52	3.69
242835	0.30	1.96	0.28	0.52	29.34	0.43	8.41	0.99	42.44	7.27	6.27
242838	0.20	1.22	─	0.50	25.86	0.39	10.70	3.34	37.29	11.48	5.52
242839	0.45	7.18	1.82	2.35	42.52	1.51	8.90	1.76	20.56	5.83	3.78
242840	0.30	3.10	0.47	1.19	31.71	0.89	9.88	2.01	34.94	8.86	4.18
242841	0.30	5.17	0.97	1.03	31.86	0.70	8.18	─	40.01	5.46	4.19
242842	0.35	4.44	0.92	1.43	30.76	0.95	9.04	1.63	38.42	4.99	3.39
242844	0.30	6.49	1.25	1.20	29.33	0.77	4.82	0.97	39.70	5.20	3.72
242823	0.38	3.63	1.01	1.32	30.60	0.96	8.81	0.58	38.75	8.12	3.55
002_ATH	0.31	8.09	1.74	2.21	39.45	1.61	7.46	1.41	23.96	4.27	3.68
003_ATH	0.23	3.37	0.81	0.50	34.40	1.08	10.93	2.13	31.91	5.80	5.86
004_ATH	0.25	2.55	0.56	0.54	28.69	0.47	10.31	2.24	37.59	7.04	6.97
007_ATH	0.40	1.32	─	0.55	28.33	0.45	10.98	2.88	36.38	9.75	5.42
009_ATH	0.35	3.84	0.81	1.39	37.90	1.22	10.16	2.07	29.29	4.59	4.54
010_ATH	0.30	3.42	0.69	0.88	33.88	1.04	10.07	1.66	34.47	5.67	3.99
012_ATH	0.20	11.13	2.24	1.97	38.74	1.40	7.63	1.05	24.71	3.20	3.37
013_ATH	0.40	3.75	0.73	1.35	31.09	0.70	8.06	1.63	35.21	5.42	2.61
014_ATH	0.28	2.81	0.77	1.18	30.73	0.47	8.40	2.29	40.35	7.92	3.55
015_ATH	0.22	3.60	0.66	1.36	31.35	0.90	10.53	1.76	32.61	8.41	4.94
017_ATH	0.33	4.34	1.02	1.86	39.84	1.44	9.11	0.88	26.58	6.55	3.29
019_ATH	0.31	3.73	0.65	1.46	33.52	1.04	10.34	1.83	25.74	5.28	5.99
Mean	0.35	5.76	1.24	1.62	35.16	1.11	8.78	1.38	31.08	5.91	3.67
SE (±)	0.01	0.33	0.07	0.07	0.56	0.05	0.15	0.08	0.80	0.24	0.13
Minimum	0.18	0.91	0.00	0.00	23.93	0.37	4.82	0.00	14.81	3.01	2.10
Maximum	0.55	12.97	2.75	2.82	45.61	1.86	11.29	3.34	42.44	11.48	6.97

*Note:* CC = chemical compound: CC1 = α-thujene, CC2 = α-pinene, CC3 = β-pinene, CC4 = ρ-cymene, CC5 = *d*-limonene, CC6 = *trans*-4-methoxythujane, CC7 = terpinen-4-ol, CC8 = thymoquinone, CC9 = carvacrol, and CC10 = longifolene.

Abbreviations: EOC = essential oil content and SE = standard error.

**Table 4:** Comparison of mean performances of 5% of the best-performed landraces selected for 10 major volatile compounds over the mean performance of improved varieties.

No.	Chemical compound	Mean values		A comparative advantage over mean values of improved varieties (%)
		Top 5% landraces	Improved varieties	
1.	α-Thujene	12.03	5.33	125.70
2.	α-Pinene	2.55	1.18	116.10
3.	β-Pinene	2.7	1.66	62.65
4.	ρ-Cymene	45.12	33.86	33.25
5.	*d*-Limonene	1.77	0.95	86.32
6.	*trans*-4-Methoxythujane	11.17	8.84	26.36
7.	Terpinen-4-ol	2.95	1.17	152.14
8.	Thymoquinone	41.51	33.12	25.33
9.	Carvacrol	10.15	6.65	52.63
10.	Longifolene	6.41	3.17	102.21

**Table 5:** Clustering of 64 *N. sativa* genotypes of Ethiopia into two clusters using mean of 10 major volatile compounds.

Cluster	Number of genotypes	Genotypes included
I	56 (87.50%)	242840, 230040, Silingo, 242823, 19884, 015_ATH, 242528, 013_ATH, 230777, 014_ATH, 240403, DERSHYE, Qeneni, 242826, 90506, ADEN, 242224, Darbera, 223072, 9067, 242842, 242833, 010_ATH, 242841, 90503, 242844, 004_ATH, 242835, 019_ATH, 009_ATH, 003_ATH, 007_ATH, 242838, 9068, 012_ATH, 90504, 229808, 002_ATH, Kena, 240404, 212520, 230039, 219970, 223069, Gammachis, Sooressaa, 017_ATH, 230037, 208688, 230038, 242223, 237989, 242225, 223070, 90502, and 215319
II	8 (12.50%)	8502, 90501, 242221, 242222, 242825, 9069, 90510, and 242839

**Table 6:** Mean value of 10 major volatile compounds of the groups of Ethiopian *N. sativa* genotypes.

No.	Compounds	Cluster	
		I	II
1.	α-Thujene	4.67	8.75
2.	α-Pinene	1.14	1.92
3.	β-Pinene	1.57	2.37
4.	ρ-Cymene	34.43	42.58
5.	*d*-Limonene	1.04	1.55
6.	*trans*-4-Methoxythujane	8.89	8.53
7.	Terpinen-4-ol	1.52	1.15
8.	Thymoquinone	32.84	20.64
9.	Carvacrol	5.49	4.88
10.	Longifolene	3.51	3.36

**Table 7:** Clustering of 64 *N. sativa* genotypes based on collection groups.

Groups	Cluster		Total
	I	II	
Amhara	13	4	24
Oromia	14	4	18
Tigray	6	—	6
SNNP	4	—	4
B/Gumuz	4	—	4
Improved	8	—	8
Total	56	8	64

Abbreviations: B/Gumuz = Benishangul-Gumuz and SNNP = Southern Nations, Nationalities, and People.

## Data Availability

All the data were included within the manuscript.
